# Eleutheroside B Ameliorates Cardiomyocytes Necroptosis in High-Altitude-Induced Myocardial Injury via Nrf2/HO-1 Signaling Pathway

**DOI:** 10.3390/antiox14020190

**Published:** 2025-02-07

**Authors:** Huxinyue Duan, Yue Han, Hongying Zhang, Tianyue Zhou, Chunjie Wu, Zhenxing Wang, Yacong He

**Affiliations:** 1 State Key Laboratory of Southwestern Chinese Medicine Resources, School of Pharmacy, Chengdu University of Traditional Chinese Medicine, Chengdu 611137, China; duanhxy@stu.cdutcm.edu.cn (H.D.); hanyue@stu.cdutcm.edu.cn (Y.H.); zhonghongying@stu.cdutcm.edu.cn (H.Z.); 13540806216@163.com (T.Z.); 2 Innovative Institute of Chinese Medicine and Pharmacy—Academy for Interdiscipline, Chengdu University of Traditional Chinese Medicine, Chengdu 611137, China; 3 School of Clinical Medicine, Chengdu University of Traditional Chinese Medicine, Chengdu 610075, China; wangzhenxing@cdutcm.edu.cn

**Keywords:** eleutheroside B, high-altitude-induced myocardial injury, necroptosis, Nrf2

## Abstract

This study was designed to evaluate the protective effects of eleutheroside B (EB) in high-altitude-induced myocardial injury (HAMI) and to unravel the underlying molecular mechanisms. SD rats were used for in vivo experiments. Following pretreatment with EB, the SD rats were exposed to a hypobaric environment within a hypobaric chamber for 48 h. Electrocardiograms, H&E staining, and serum biochemical indices were measured to evaluate the protective effects of EB on HAMI. Immunofluorescence and Western blotting were utilized to detect the expression of associated proteins. In parallel, a hypobaric hypoxic cell incubator was used to establish an in vitro model of hypobaric hypoxia-induced cell injury. The anti-necroptotic effect and its potential underlying mechanisms were investigated and verified in vitro. Exposure to hypobaric hypoxia led to electrocardiogram disorders, pathological changes in myocardial tissue, increased concentrations of BNP and CK-MB, and elevated levels of oxidative stress indicators and inflammatory factors. Additionally, the expression of necroptosis-related proteins was upregulated. Pretreatment with EB effectively ameliorated myocardial injury caused by hypobaric hypoxia, mitigated oxidative stress and inflammation, and suppressed necroptosis. Furthermore, EB facilitated the translocation of Nrf2 into the nucleus. In conclusion, this study provides evidence suggesting that EB may exert a protective effect against HAMI by inhibiting cardiomyocyte necroptosis via the Nrf2/HO-1 signaling pathway.

## 1. Introduction

In recent years, owing to expanding opportunities in work, study, and tourism, an escalating number of individuals are migrating from low-altitude to high-altitude regions. Generally, an area with an altitude exceeding 2500 m above sea level is classified as a high-altitude area [1]. High-altitude areas are typified by low atmospheric pressure and oxygen scarcity [2]. Upon acute exposure to high-altitude conditions, a series of acute mountain sickness (AMS) symptoms, including dizziness, nausea, and arrhythmia appear [3,4]. Recent studies have shown that high-altitude exposure affects cardiovascular function. Initially, there is an augmentation in heart rate, myocardial contractility, and cardiac output. While these physiological responses serve as compensatory mechanisms, they may ultimately inflict severe damage on the heart, potentially culminating in heart failure and cardiac mortality [5,6,7]. It has also been reported that hypobaric hypoxia can induce a spectrum of cardiac pathologies, including impaired cardiac function, cardiomyocyte apoptosis, heightened oxidative stress, and elevated inflammatory factors [8]. Nevertheless, the underlying mechanisms of HAMI remain unclear. This lack of clarity presents substantial challenges for the advancement of medical strategies aimed at preventing and treating HAMI.

Previous studies have demonstrated that exposure to hypobaric hypoxia results in an elevation of reactive oxygen species (ROS) levels [9,10]. Excessive accumulation of ROS cannot be compensated for by antioxidant enzymes in vivo, causing oxidative stress and cell death [11,12]. Unregulated oxidative stress has been implicated in promoting the development of hypobaric hypoxia-induced pathology [13]. Excessive ROS have the capacity to induce necroptosis, a form of programmed cell death that exhibits characteristics of both necrosis and apoptosis and is governed by receptor-interacting protein (RIP) kinases. In brief, upon activation, RIPK1 is recruited, and this process is accompanied by the downregulation of caspase-8. RIPK1 subsequently interacts with RIPK3 to form a necrosome complex with mixed lineage kinase domain-like pseudokinase (MLKL), which is then phosphorylated by RIPK3. The phosphorylation of MLKL leads to membrane rupture and eventually, cell death [14].

Nrf2 is a transcription factor that belongs to the Cap’n’collar-BZIP family. Numerous studies have demonstrated that Nrf2 plays a crucial role in cellular protection through its regulatory function in oxidative stress [15]. Nrf2 forms a complex with Kelch-like epichlorohydrin-related protein (Keap1) under non-oxidative stress conditions. In the presence of oxidative stress, Nrf2 undergoes phosphorylation, which triggers its translocated into the nucleus, where it forms heterodimers (Nrf2-Msf) with Maf proteins and Jun bZip transcription factors. The transcription process of Nrf2 is subsequently initiated, followed by the regulation of downstream gene expression to regulate oxidative stress-related pathways [16]. Previous reports have indicated that upregulation of Nrf2 expression can effectively inhibit necroptosis [17]. Moreover, activation of proteins associated with the Nrf2 signaling pathway has been shown to significantly ameliorate myocardial injury caused by hypoxia and oxidative stress [18]. Despite these findings, the role of Nrf2 in HAMI remains poorly understood.

Current pharmacological interventions for mitigating high-altitude-induced sickness include acetazolamide, dexamethasone, and nifedipine; however, their efficacy in treating HAMI remains unclear. These medicines may elicit side effects, such as allergic reactions, headache, and chest pain [19]. Consequently, developing effective, safe, and accessible medicines to improve HAMI is important. *Acanthopanax senticosus* (Rupr. et Maxim.) Harms (AS), an economic crop with both medicinal and edible properties, is commonly employed in the prevention of high-altitude sickness [10]. Eleutheroside B (EB), the principal bioactive constituent of AS, exhibits anti-inflammatory, antioxidant, and antidepressant properties [5,20,21]. In 2021, Zhang et al. investigated the effects of EB on cardiac electrophysiology and its role in suppressing atrial fibrillation. They found that EB could inhibit late sodium current and transient sodium current, but had no effect on other currents or action potential duration. EB was also shown to suppress arrhythmias by blocking the late sodium current [22]. Furthermore, EB can mediate Nrf2 nuclear translocation [23]. Despite these promising findings, the protective effects of EB on HAMI have not yet been clarified. Herein, we hypothesize that inhibiting necroptosis via Nrf2-antioxidant response signaling may have a protective effect on HAMI.

## 2. Materials and Methods

### 2.1. Chemicals and Reagents

Eleutheroside B (HPLC ≥ 98%) was supplied by Chengdu Manster Biotechnology Co. (Chengdu, China). Commercial ELISA kits for glutathione (GSH), malondialdehyde (MDA), interleukin-1β (IL-1β), and tumor necrosis factor-α (TNF-α) were obtained from Ruixin Biotechnology Co. (Quanzhou, China). Antibodies against Heme Oxygenase 1 (HO-1, A11102), Receptor-interacting protein kinase 1 (RIPK1, A7414), Receptor-interacting protein kinase 3 (RIPK3, A5431), Mixed lineage kinase domain-like pseudo kinase (MLKL, A21894), Phospho-MLKL(AP0949), Nuclear factor erythroid 2-related factor 2 (Nrf2, A0674), and β-actin (AC026) and goat anti-rabbit IgG (AS070) were purchased from ABclonal Biotechnology Co. (Wuhan, China). ML385 was purchased from MedChemExpress (Shanghai, China). A reactive oxygen species fluorometric assay kit was obtained from Beyotime Co. (Shanghai, China).

### 2.2. Animals and In Vivo HH Model Establishment

Male Sprague–Dawley rats (160–180 g, 6–8 weeks old) were obtained from Sibeifu Biotechnology Co., Ltd. (Beijing, China). This study was approved by the Medical Ethics Committee of Cheng University of Traditional Chinese Medicine, China (Grant No. 2023386). The Rats were maintained under standard conditions (22 ± 1 °C, 55 ± 5 humidity, 12 h light, and dark cycle), with free access to standard diet and water.

The HH rat model was established using a hypobaric chamber following a previously described protocol [5]. Briefly, the rats were placed in a hypobaric chamber, accompanied by a supply of water and food. The altitude was then set to 6000 m (corresponding to an atmospheric pressure of approximately 9.6 kPa), with an elevation rise rate of 20 m/s. The total processing time was 48 h.

### 2.3. Animal Grouping and Treatments

Following a 7-day adaptive feeding period, 30 rats were randomly allocated into five experimental groups, with six rats in each group. The groups were as follows: (1) the control group (Con), (2) the hypobaric hypoxia group (HH), (3) the hypobaric hypoxia with low-dose EB group (EB-L + HH, 50 mg/kg), (4) the hypobaric hypoxia with high-dose EB group (EB-H + HH, 100 mg/kg), and (5) the hypobaric hypoxia with dexamethasone group (Dex + HH, 4 mg/kg). The drug dosages were selected based on previous studies [5,10]. Dexamethasone served as a positive control. EB and dexamethasone were dissolved according to a previously described method [10]. The rats were pretreated with the corresponding medications via intraperitoneal injection for three consecutive days. The rats in the Con group and the HH group were injected with the solvent used for drug preparation. After the final administration, all rats except those in the Con group were placed in a hypobaric chamber. The chamber was calibrated to simulate an altitude of 6000 m, and the rats were exposed to these hypobaric hypoxia conditions for a duration of 48 h.

Upon completion of the hypobaric hypoxia exposure, the rats were anesthetized with sodium pentobarbital. Immediately thereafter, an electrocardiogram (ECG) was recorded for each rat. Blood samples were obtained from the abdominal aorta of rats, followed by serum collection through centrifugation for the purpose of detecting biochemical indicators. The hearts were excised from the rats for further analysis.

### 2.4. Cell Culture and In Vitro HH Model Establishment

We employed SD rats for in vivo experiments. Therefore, rat cardiomyocyte-like H9c2 cells were used for in vitro experiments. The H9c2 cells were purchased from Yubo Biotechnology Co., Ltd. (Shanghai, China). DMEM supplemented with 10% fetal bovine serum (FBS) and 1% penicillin-streptomycin was used to maintain H9c2 cells in culture. Cultivation was conducted in a controlled environment within an incubator set at 37 °C with 5% CO_2_. Four- to ten-generation cells were used in this study.

A hypobaric hypoxia cell incubator (Tawang Technologies, Shanghai, China) was used to simulate the plateau environment and establish the HH injury cell model. Specifically, we fixed the height at 6000 m (47 kPa, consistent with the HH animal model), oxygen content at 1% [24], and carbon dioxide content at 5%. To determine the most appropriate conditions for the model establishment, a series of intervention times (2, 4, 6, 8, 10, 12, and 24 h) were systematically evaluated. Assays for measuring cell viability and evaluating cell injury were performed using a CCK-8 assay.

### 2.5. Cell Viability Assay

To determine the appropriate dosage of EB for intervention, a CCK-8 assay was used to assess the viability of H9c2 cells. Initially, H9c2 cells were seeded in 96-well plates and allowed to adhere fully. Subsequently, the cells were exposed to a range of EB concentrations for a 24 h period. After the 24 h incubation with EB, the medium was replaced with fresh medium containing the CCK-8 reagent, and the cells were further incubated for an additional 30 min. Cell viability was calculated by measuring the optical density (OD) of each well using a microplate reader (Bio-Rad, Hercules, CA, USA) at 450 nm.

### 2.6. ECG Recording

After exposure to hypobaric hypoxia for 48 h, the rats were anesthetized and then immobilized in a supine position. Three electrocardiograph electrodes (Chengdu Techman Software Co., Ltd., Chengdu, China) were successively connected to the right upper limb, right lower limb, and left lower limb of each rat. Cardiac function-related data, including heart rate, QT interval, QTc interval, T-wave amplitude, R-wave amplitude, and P-wave amplitude, were recorded and analyzed using a BL-420 N system (Chengdu Techman Software Co., Ltd., Chengdu, China).

### 2.7. Histopathology of Heart Tissues

After the ECG recording was completed, all the rats were euthanized. Their cardiac tissues were excised and rinsed with saline solution. After fixation in 4% paraformaldehyde, dehydration in ethanol solutions, and clarification in xylene, the myocardial specimens were embedded in paraffin wax. The tissues were subsequently sectioned into 5 μm-thick slices. These slices were then mounted on glass slides and baked to ensure proper adhesion. Prior to staining, the paraffin-embedded tissue sections were deparaffinized in xylene and rehydrated through ethanol solutions. The rehydrated sections were then stained with hematoxylin and eosin. Following dehydration and sealing, the specimens were examined under a light microscope.

### 2.8. Determination of Cardiac Biomarkers, Antioxidant Status, and Inflammatory Cytokines

The collected serum samples were utilized for the quantification of relevant biochemical indicators, including CK-MB, BNP, GSH, MDA, IL-1β, and TNF-α. The detection of these biochemical markers was carried out using commercially available kits, following the manufacturers’ instructions.

### 2.9. TUNEL Assay

Cell apoptosis in myocardial tissues was assessed using a one-step TUNEL apoptosis assay kit (Beyotime Biotechnology, Shanghai, China). In accordance with the protocol, the paraffin-embedded myocardial tissue sections underwent a series of pre-treatment steps. Subsequently, the sections were incubated with the TUNEL reaction mixture at 37 °C for one hour. After that, they were subjected to counterstaining with DAPI. Finally, a confocal laser microscope was used to observe the positive TUNEL signal within the specimens.

### 2.10. Immunofluorescence Staining

As previously described, following preparation, heart sections were incubated overnight at 4 °C with the Nrf2 primary antibodies. Subsequently, the sections were incubated with secondary antibodies for 1 h at room temperature. After the secondary antibody incubation, the sections were stained with DAPI, and observed under a fluorescence microscope.

After being fixed in 4% formaldehyde for 20 min, the cells were washed and incubated with 0.3% Triton for 30 min. A blocking step was then performed with serum for 1 h, followed by incubation with an Nrf2 antibody overnight at 4 °C. Subsequently, a fluorescently labeled secondary antibody was added for 1 h at room temperature. After the cells were washed with TBST, DAPI solution was added, and a laser confocal microscope was used to observe the cells.

### 2.11. Flow Cytometry for ROS and Necroptosis

The detection of ROS was conducted using a ROS assay kit obtained from Beyotime Biotechnology (Shanghai, China). H9c2 cells were harvested and suspended in DCFH-DA (10 μM). The cell suspension was then incubated at 37 °C for a duration of 20 min. Following the incubation period, the cells were washed three times with serum-free culture medium. The intracellular ROS levels were then assessed using flow cytometry (BD Company, New York, NY, USA).

An Annexin V-FITC/PI cell death assay kit was employed to detect necroptosis. In a systematic manner, H9c2 cells were collected and washed with PBS. The cells were suspended in Annexin V-FITC binding buffer and Annexin V-FITC was added to the cell suspension. After that, PI was added and the solution was mixed. The mixture was incubated at room temperature for 20 min, after which flow cytometry (BD Company, NY, USA) was used to detect the cells.

### 2.12. Western Blot

A previously reported method was used for Western blotting [5]. Proteins were separated using SDS-PAGE, then target proteins were blotted on PVDF membranes with antibodies against HO-1, RIPK1, RIPK3, MLKL, and p-MLKL, followed by incubation with secondary antibodies. Finally, the target proteins were visualized with chemiluminescence. The protein bands were analyzed using Image Pro Plus 6.0 software.

### 2.13. Statistical Analysis

In this study, the results were expressed as mean ± SD. Statistical analyses were performed using one-way analysis of variance (ANOVA), followed by Tukey’s HSD test. *p* < 0.05 was used to evaluate the statistical significance of the differences.

## 3. Results

### 3.1. EB Improved HH-Induced Abnormal Electrocardiographic Signatures in Rats

**Figure 1:**
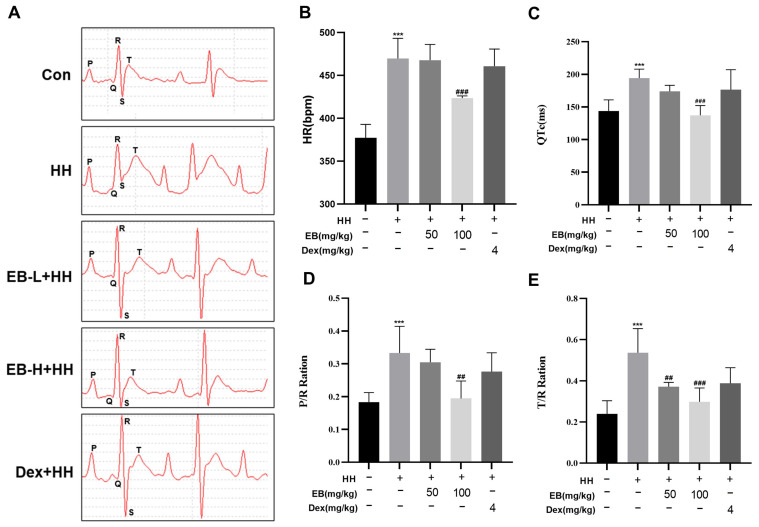
EB improved HH-induced abnormal electrocardiographic signatures in rats. All rats from five different groups were anesthetized to perform electrocardiography. (**A**) Representative electrocardiographic pattern. (**B**) Quantitative measurement of heart rate. (**C**) Quantitative measurement of corrected QT intervals. (**D**) Quantitative measurement of P/R ratio. (**E**) Quantitative measurement of T/R ratio. Quantitative results were analyzed using a BL-420N system. Values are presented as mean ± SD (*n* = 6). *** *p* < 0.001 compared with Con group; ^##^ *p* < 0.01, ^###^ *p* < 0.001 compared with HH group. Con, the control group; HH, the hypobaric hypoxia group; EB-L + HH, the hypobaric hypoxia with low-dose EB group; EB-H + HH, the hypobaric hypoxia with high-dose EB group; Dex + HH, the hypobaric hypoxia with dexamethasone group.

ECG recording was employed to assess alterations in the cardiac function of rats. As shown in Figure 1, in the HH group, significant changes were observed. Specifically, there was an elevation in heart rate, an increase in the QTc interval, and augmentation in both the ratio of T-wave amplitude to R-wave amplitude (T/R ratio) and the ratio of P-wave amplitude to R-wave amplitude (P/R ratio) (*p* < 0.001 vs. the Con group). These changes suggested that exposure to a hypobaric hypoxic environment induced tachycardia and conduction abnormalities in HH group rats. Pretreatment with the high-dose EB reversed these abnormalities. This was evidenced by a reduction in heart rate, a shortening of the QTc interval, and a decrease in the T/R and P/R ratios (*p* < 0.01 vs. the HH group). Pretreatment with the low-dose EB and Dex led to a partial reversal of the HH-induced ECG changes; however, these improvements did not reach statistical significance. These results suggested that EB improved heart function in HAMI rats.

### 3.2. EB Ameliorates Cardiac Injury in HH-Induced Rats

**Figure 2:**
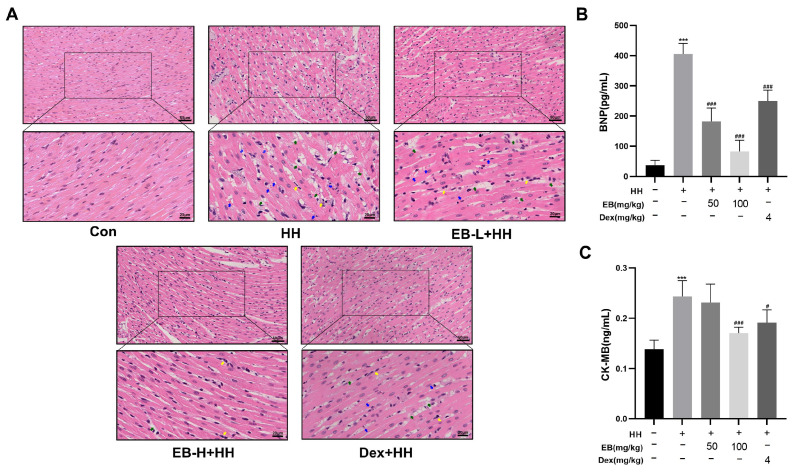
EB ameliorates cardiac injury in HH-induced rats. (**A**) Images of H&E stained rats’ myocardium. Blue arrows indicate hemorrhage; yellow arrows indicate inflammatory infiltration; green arrows indicate the breakage of myocardial fibers. (**B**) Levels of BNP in rat serum. (**C**) Levels of CK-MB in rat serum. Values are presented as mean ± SD (*n* = 6). *** *p* < 0.001 compared with Con group; ^#^ *p* < 0.05, ^###^ *p* < 0.001 compared with HH group. Con, the control group; HH, the hypobaric hypoxia group; EB-L + HH, the hypobaric hypoxia with low-dose EB group; EB-H + HH, the hypobaric hypoxia with high-dose EB group; Dex + HH, the hypobaric hypoxia with dexamethasone group.

The myocardium was stained with H&E to evaluate pathological damage. As illustrated in Figure 2A, in the Con group, the myocardial tissue of the rats exhibited a well-organized structure. The myofibrils and nuclei presented normal morphological characteristics. Exposure to hypobaric hypoxia resulted in structural disorders of myocardial tissue in rats. These pathological changes included space widening, rupture, microbleeds, and nuclear pyknosis. Moreover, inflammatory cell infiltrates were observed. Interestingly, pretreatment with EB and Dex ameliorated the above-described myocardial tissue injury. Both low-dose and high-dose EB exerted protective effects on myocardial tissue. And high-dose EB demonstrated superior efficacy compared to low-dose EB. Furthermore, the improvement in myocardial tissue morphology caused by high-dose EB was comparable to that caused by Dex.

Serum levels of cardiac biomarkers can, to a certain extent, mirror the severity of myocardial injury [25]. CK-MB is a major creatine kinase isoenzyme, which is present in the myocardium, skeletal muscle, and brain. It is worth noting that a high concentration of CK-MB is specific to the myocardium. Upon myocardial cell death, CK-MB is quickly released, leading to a rapid concentration increase in the bloodstream [26]. BNP is a member of the natriuretic peptides family and serves as an effective biomarker for the diagnosis of myocardial dysfunction. Tissue hypoxia can trigger an acute surge in blood BNP levels [27]. Therefore, the serum levels of BNP and CK-MB were determined using ELISA kits. The results showed that the rats in the HH group had higher serum levels of CK-MB and BNP compared to those in the Con group (*p* < 0.001). This finding implies that exposure to hypobaric hypoxia exerts detrimental effects on the rat hearts. Conversely, in all the drug-intervention groups, the serum concentration of CK-MB and BNP were decreased. Among them, the EB-H group showed the lowest levels of these two biomarkers when compared with the HH group (Figure 2B,C).

### 3.3. EB Improved HH-Induced Oxidative Stress and Inflammation

**Figure 3:**
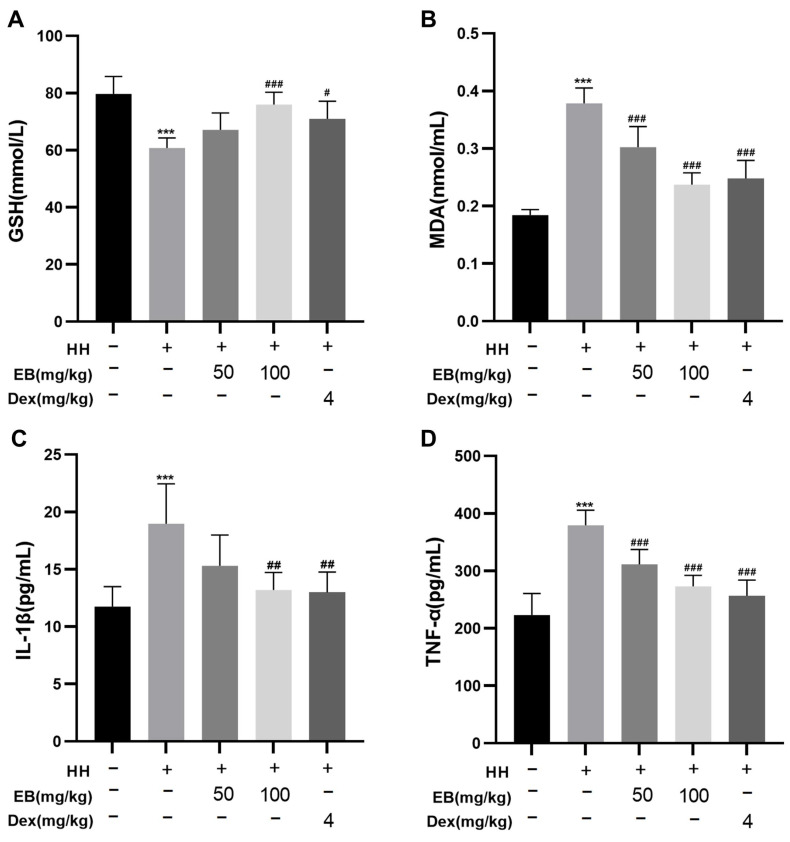
EB ameliorated HH-induced oxidative stress and inflammation. (**A**) The concentration of GSH in serum. (**B**) The concentration of MDA in serum. (**C**) The concentration of IL-1β in serum. (**D**) The concentration of TNF-α in serum. Values are presented as mean ± SD (*n* = 6). *** *p* < 0.001 compared with Con group; ^#^ *p* < 0.05, ^##^ *p* < 0.01, ^###^ *p* < 0.001 compared with HH group. Con, the control group; HH, the hypobaric hypoxia group; EB-L + HH, the hypobaric hypoxia with low-dose EB group; EB-H + HH, the hypobaric hypoxia with high-dose EB group; Dex + HH, the hypobaric hypoxia with dexamethasone group.

According to previous studies, hypobaric hypoxia causes oxidative stress, which may exacerbate damage to the organism [25]. In addition, oxidative stress has been shown to mediate necroptosis [28]. Therefore, we aimed to quantify the level of oxidative stress in rat serum. As shown in Figure 3A, the level of GSH, an antioxidant enzyme, was significantly lower in the HH group compared to the Con group (*p* < 0.001). However, pretreatment with Dex and high-dose EB led to a significant increase in GSH levels (*p* < 0.05). In addition, hypobaric hypoxia resulted in a significant elevation of MDA (Figure 3B) in the rat serum (*p* < 0.001). Pretreatment with Dex and EB (50 and 100 mg/kg) significantly reduced the MDA content (*p* < 0.001). The above results showed that EB could ameliorate oxidative stress caused by hypobaric hypoxia.

Previous studies have suggested that necroptosis may play a crucial role in promoting the onset and progression of inflammation, leading to elevated levels of inflammatory factors [29]. In light of this, we detected the concentrations of inflammatory factors in the serum. As shown in Figure 3C,D, the concentrations of IL-1β and TNF- α in the HH group were increased (*p* < 0.001 vs. the Con group). Conversely, intervention with Dex and EB significantly mitigated the increase in IL-1β and TNF-α concentrations. Notably, high-dose EB exhibited a more pronounced anti-inflammatory effect compared to low-dose EB. These results demonstrated that EB could alleviate the inflammatory response induced by hypobaric hypoxia.

### 3.4. EB Inhibited Necroptosis in HH-Induced Rats

**Figure 4:**
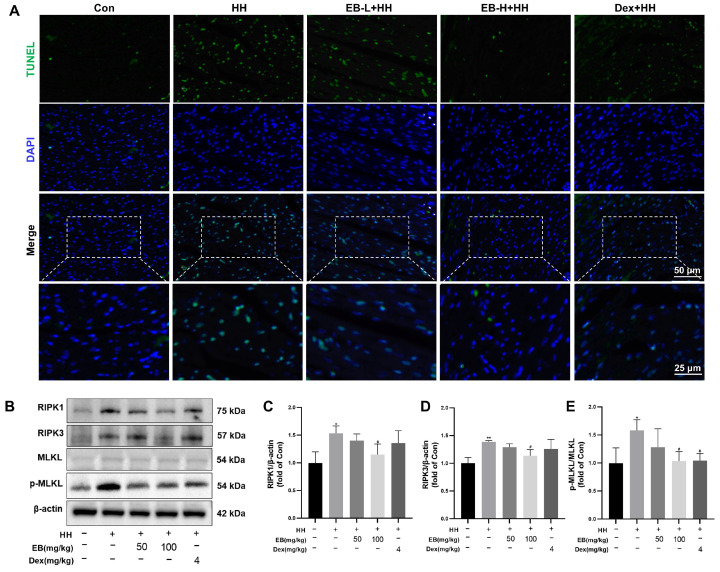
EB inhibited necroptosis in HH-induced rats. (**A**) Representative images of TUNEL staining. Green fluorescence indicated TUNEL-positive nuclei, while blue fluorescence indicated total nuclei. Bar = 50 μm. (**B**) Western blot analysis of the expression of necroptosis-related proteins. (**C**–**E**) The quantification of RIPK1, RIPK3, and p-MLKL/MLKL in rats’ heart tissue. Values are presented as mean ± SD (*n* = 3). * *p* < 0.05, ** *p* < 0.01 compared with Con group; ^#^ *p* < 0.05 compared with HH group. Con, the control group; HH, the hypobaric hypoxia group; EB-L + HH, the hypobaric hypoxia with low-dose EB group; EB-H + HH, the hypobaric hypoxia with high-dose EB group; Dex + HH, the hypobaric hypoxia with dexamethasone group.

Tissue injury is closely correlated with cell death. Given that extensive apoptosis is one of the important characteristics of necroptosis [30,31], we employed TUNEL fluorescence staining to assess the apoptosis level in the myocardial tissues. As shown in Figure 4A, a significantly higher number of TUNEL-positive cells were observed in the myocardial tissues of the HH group compared to those of the Con group. Conversely, when EB and Dex were administered for intervention, the apoptotic trend was reversed.

The expression of proteins associated with necroptosis was detected using Western blotting. The results (Figure 4B–E) indicated that compared with Con group rats, hypobaric hypoxia significantly increased the expression of RIPK1 and RIPK3, as well as the p-MLKL/MLKL ratio. Conversely, the expression of RIPK1, RIPK3, and p-MLKL/MLKL decreased in the EB-L + HH group and EB-H + HH group, with the EB-H + HH group showing a more significant effect (*p* < 0.05 vs. the HH group). These results suggested that pretreatment with EB suppressed HH-induced necroptosis in myocardial tissue.

### 3.5. EB Facilitated Nrf2 Translocation to the Nucleus and Upregulated the Expression of HO-1

**Figure 5:**
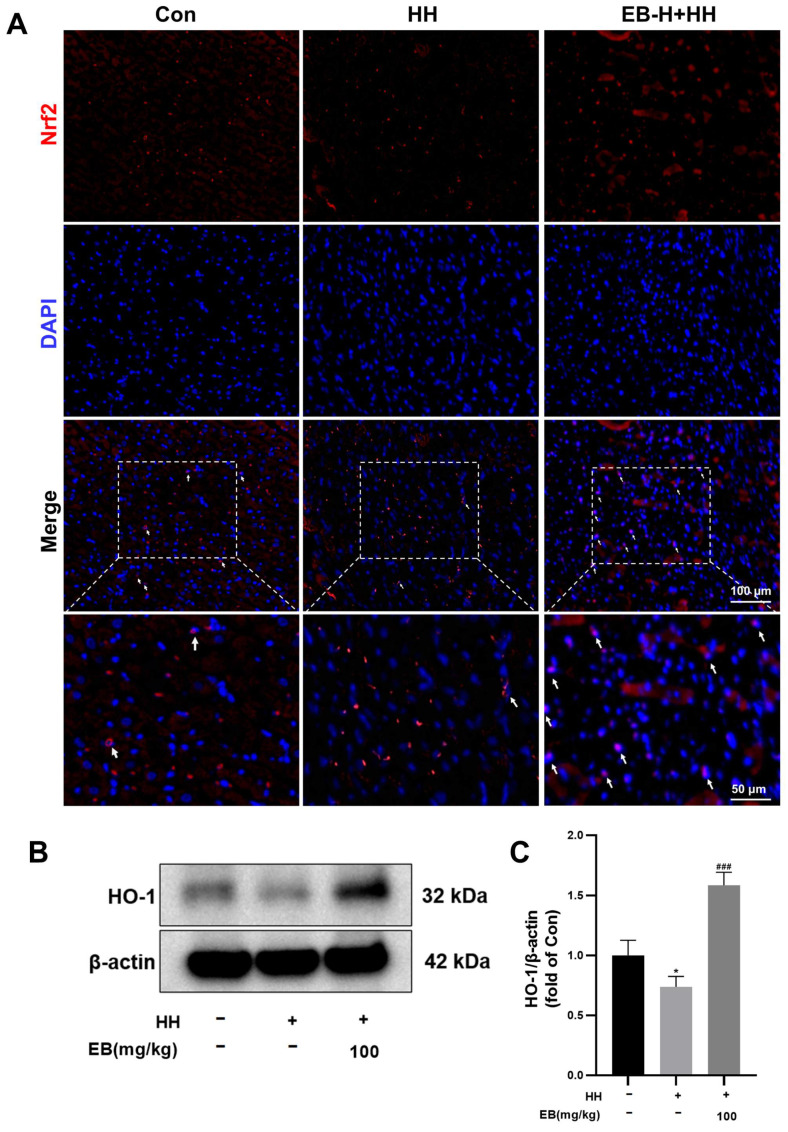
EB facilitated Nrf2 translocation to the nucleus and upregulated the expression of HO-1. (**A**) Representative immunofluorescence images demonstrating nuclear translocation of Nrf2 in heart tissue. White arrows indicate the Nrf2 fluorescence signal colocalized with DAPI staining. Bar = 100 μm. *n* = 3. (**B**) Western blot analysis of the expression of HO-1. (**C**) The quantification of HO-1 protein in rats’ heart tissue. Values are presented as mean ± SD (*n* = 3). * *p* < 0.05 compared with Con group; ^###^ *p* < 0.001 compared with HH group. Con, the control group; HH, the hypobaric hypoxia group; EB-L + HH, the hypobaric hypoxia with low-dose EB group; EB-H + HH, the hypobaric hypoxia with high-dose EB group; Dex + HH, the hypobaric hypoxia with dexamethasone group.

Nrf2 is known to play a crucial role in the regulation of oxidative stress and also exerts a regulatory influence on necroptosis. The immunofluorescence assay (Figure 5A) revealed that the translocation of Nrf2 into the nucleus was reduced in the HH group compared to the Con group. In contrast, pretreatment with EB (100 mg/mL) significantly increased Nrf2 accumulation in the nucleus. Nrf2 exerts its antioxidant stress functions by the regulation of HO-1, which in turn modulates the activity of antioxidant enzymes. WB was employed to quantify the expression of HO-1. The results indicated that compared to the HH group, EB pretreatment significantly upregulated the expression of HO-1. These results suggested that under hypobaric hypoxic conditions, EB could exert antioxidant effects via the Nrf2/HO-1 pathway.

### 3.6. EB Improved HH-Induced H9c2 Cell Viability and Prevented HH-Induced Oxidative Stress In Vitro

**Figure 6:**
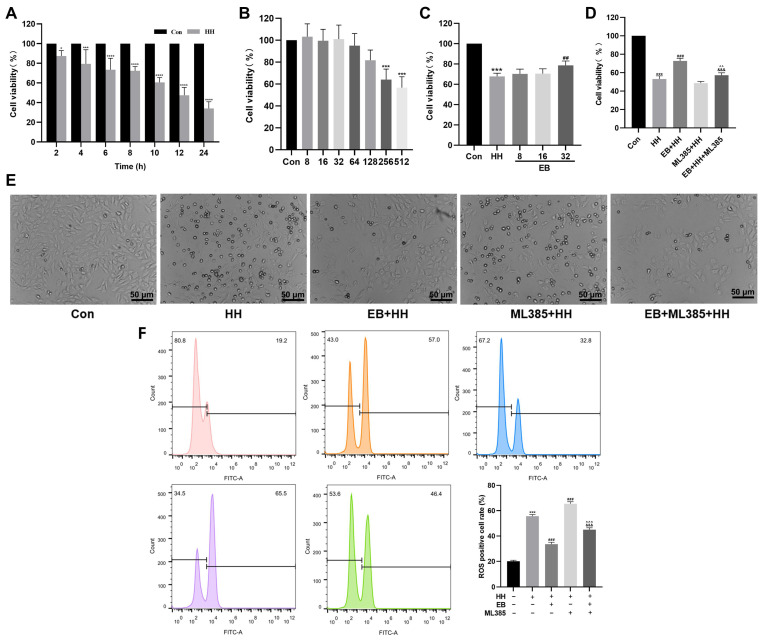
Effects of EB and ML385 on cell viability and ROS levels in HH-induced H9c2 cells. (**A**) Cell viability of H9c2 cells treated with HH. (**B**) Cell viability of H9c2 cells treated with different doses of EB. (**C**) Cell viability of H9c2 cells treated with HH and EB. (**D**) Cell viability of H9c2 cells treated with HH, EB, and Nrf2 inhibitor ML385. (**E**) Represented cell morphology of H9c2 cells. (**F**) Flow cytometry analysis of the levels of ROS in H9c2 cells. Values are presented as mean ± SD (*n* = 3). * *p* < 0.05, *** *p* < 0.001, **** *p* < 0.0001 compared with Con group; ^##^ *p* < 0.01, ^###^ *p* < 0.001 compared with HH group; ^&&&^ *p* < 0.001 compared with EB + HH group; ^^^^ *p* < 0.01, ^^^^^ *p* < 0.001 compared with ML385 + HH group. Con, the control group; HH, the hypobaric hypoxia group; EB-L + HH, the hypobaric hypoxia with low-dose EB group; EB-H + HH, the hypobaric hypoxia with high-dose EB group; Dex + HH, the hypobaric hypoxia with dexamethasone group.

To further investigate the protective effect of EB on HAMI and to confirm the role of Nrf2, an HH-induced H9c2 cell model was established. The hypoxic exposure duration that led to approximately 50% cell viability was determined and selected as the intervention time for subsequent experiments (12 h) (Figure 6A). Then, the working concentration of EB for subsequent experiments, we screened. As shown in Figure 6B, EB at concentrations of 8, 16, and 32 μM had no significant effect on the viability of H9c2 cells, and no reduction in cell viability was observed. After pretreatment with EB (8, 16, or 32 μM), H9c2 cells were exposed to HH conditions. The concentration of EB at 32 μM significantly increased cell viability (*p* < 0.01 vs. the HH group), while EB at concentrations of 8 and 16 μM had little effect on the reduction in cell viability caused by HH (*p* > 0.05 vs. the HH group).

To verify whether EB ameliorated HH-induced injury through the Nrf2/HO-1 pathway, H9c2 cells were treated with the Nrf2 inhibitor ML385. As shown in Figure 6D,E, EB notably enhanced the viability of HH-induced H9c2 cells. However, when cells were pretreated with 1 μM of ML385, the protective effect of EB was attenuated. Additionally, compared with the HH group, the ML385 + HH group did not have a significant effect on the HH model. Furthermore, the fluorescence probe DCFH-DA was used to detect ROS generation in H9c2 cells. As shown in Figure 6F, the level of intracellular ROS significantly elevated in the HH group, and EB reduced this increase. Nevertheless, ML385 impaired the ROS-reducing effect of EB.

### 3.7. EB Inhibited HH-Induced Necroptosis by Facilitating the Nuclear Translocation of Nrf2 and Upregulating HO-1

**Figure 7:**
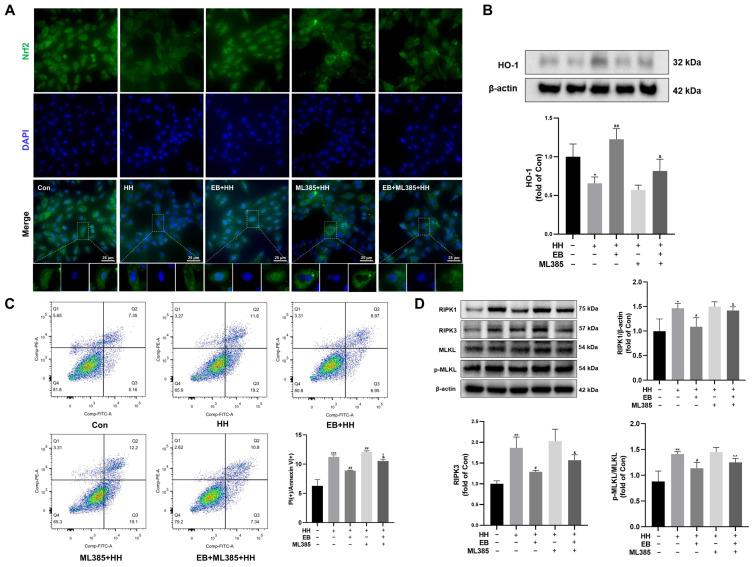
Effects of EB and ML385 on necroptosis and the Nrf2/HO-1 signaling in HH-induced H9c2 cells. (**A**) Representative images of Nrf2 expression in H9c2 cells with different treatments. (**B**) Western blot for HO-1 expression in H9C2 cells. (**C**) Quantitative analysis of apoptotic cells quantified by flow cytometry. (**D**) Western blot for RIPK1, RIPK3, MLKL, and p-MLKL expression in H9C2 cells. Values are presented as mean ± SD (*n* = 3). * *p* < 0.05, ** *p* < 0.01, *** *p* < 0.001 compared with Con group; ^#^ *p* < 0.05, ^##^ *p* < 0.01 compared with HH group; ^&^ *p* < 0.05 compared with EB + HH group; ^^^ *p* < 0.05, ^^^^ *p* < 0.01 compared with ML385 + HH group. Con, the control group; HH, the hypobaric hypoxia group; EB-L + HH, the hypobaric hypoxia with low-dose EB group; EB-H + HH, the hypobaric hypoxia with high-dose EB group; Dex + HH, the hypobaric hypoxia with dexamethasone group.

To determine whether the anti-necroptotic effect of EB on H9c2 cells induced by hypobaric hypoxia is associated with the Nrf2/HO-1 pathway, ML385 was utilized. The results are shown in Figure 7. The results of the immunofluorescence assay (Figure 7A) demonstrated that the nuclear translocation of Nrf2 was decreased by HH. Pretreatment with EB effectively promoted the nuclear translocation of Nrf2. However, ML385 reversed the effect of EB. As shown in Figure 7B, EB significantly upregulated the expression of HO-1 (*p* < 0.05 vs. the HH group). However, ML385 downregulated the expression of HO-1 (*p* < 0.05 vs. the EB + HH group).

Furthermore, flow cytometry was employed to quantify the necroptosis rate of H9c2 cells. The cells were stained with Annexin V-FITC/PI, and Annexin V^+^/PI^+^ cells were defined as necroptotic or late apoptotic cells. As illustrated in Figure 7C, the percentage of Annexin V^+^/PI^+^ cells increased after HH exposure. However, pretreatment with EB effectively reduced this percentage. Notably, the percentage of Annexin V^+^/PI^+^ cells was significantly higher in the EB + ML385 + HH group compared to the EB + HH group. Interestingly, the alterations in the expression of necroptosis-related proteins across different groups were consistent with the flow cytometry results. As shown in Figure 7D, the expression of RIPK1, RIPK3, and the ratio of p-MLKL/MLKL were downregulated by EB, compared with those in the HH group. Moreover, the addition of ML385 weakened the inhibitory effect of EB on the expression of these necroptosis-related proteins.

## 4. Discussion

The results of our research indicated that hypobaric hypoxia exerted detrimental effects on the rat hearts. These adverse impacts were manifested in multiple aspects, including abnormal electrocardiogram outcomes, myocardial tissue damage, elevated concentrations of myocardial injury markers, and increased concentrations of oxidative stress indicators and inflammatory factors. Moreover, necroptosis was detected in both HH-induced rats and H9c2 cells. In this study, EB was reported for the first time to have a protective effect on HH-induced HAMI. EB improved the electrocardiogram and pathological changes in myocardial tissue in rats, and reduced the concentration of BNP and CK-MB, demonstrating a protective effect on the heart. Furthermore, EB pretreatment resulted in a decrease in the concentrations of MDA, IL-1β, and TNF-α, while simultaneously increasing the concentration of GSH. Further experimental investigations revealed that EB downregulated the expression of RIPK1, RIPK3, MLKL, and p-MLKL in both HH-induced rats and H9c2 cells. More importantly, EB promoted Nrf2 translocation to the nucleus and upregulated the expression of HO-1. Collectively, these results suggested that EB might be a potential therapeutic compound for protecting against HAMI by activating the Nrf2/HO-1 signaling pathway to inhibit necroptosis.

Necroptosis is a form of programmed cell death regulated by RIP kinases. The activation of these kinases ultimately leads to phosphorylation of MLKL and subsequent rupture of the cell membrane. Necroptosis has been observed in hypoxia/reoxygenation-injured hearts, diabetic cardiomyopathy, and doxorubicin-induced myocardial injury [30,32,33]. In our study, increased expression of RIPK1, RIPK3, MLKL, and p-MLKL was observed in both HH-induced rats and H9c2 cells. This finding strongly suggested that necroptosis may be one of the key underlying mechanisms contributing to HH-induced HAMI. Notably, EB alleviated HH-induced necroptosis by downregulating the expression of RIPK1, RIPK3, MLKL, and p-MLKL.

Hypobaric hypoxic conditions are known to induce oxidative stress and organ dysfunction through the generation and accumulation of reactive oxygen species (ROS) [34]. In our investigation, we observed a significant decrease in the serum level of GSH, an important antioxidant enzyme, in rats exposed to hypobaric hypoxia. Concurrently, there was a substantial increase in the level of MDA, a biomarker of oxidative damage. Interestingly, EB pretreatment reversed these changes, thereby alleviating oxidative stress. Oxidative stress is recognized as one of the key triggers of necroptosis. It activates critical proteins involved in the necroptotic pathway, including RIPK1, RIPK3, and MLKL. The phosphorylation of MLKL mediates the destruction of the plasma membrane, allowing the release of cellular contents. Subsequently, the release of damage-associated molecular patterns from cells initiates an inflammatory process by binding to receptors on the surface of innate immune cells [35]. Our results showed that the expression of necroptosis-related proteins (RIPK1, RIPK3, and p-MLKL/MLKL) was upregulated in the myocardial tissue of the rats in the HH group. Simultaneously, the serum levels of inflammatory factors (IL-1β and TNF-α) were also increased. However, pretreatment with EB reduced the levels of IL-1β and TNF-α, as well as downregulated the expression of RIPK1, RIPK3, and p-MLKL/MLKL. These results suggested that EB could ameliorate HH-induced cardiac injury, at least in part, by suppressing necroptosis.

Previous research has indicated that regulating Nrf2 exerts an influence on necroptosis [36]. Nrf2 serves as a crucial component of the endogenous antioxidant system and plays a protective role in counteracting oxidative stress and preventing cell death in cardiovascular diseases [37,38,39,40,41]. Interestingly, the findings of our study demonstrate that EB promoted the translocation of Nrf2 from the cytoplasm to the nucleus. This phenomenon represented the underlying molecular mechanism through which EB ameliorates HAMI. To further verify this hypothesis, we established an in vitro HH-induced H9c2 cell model for subsequent research. The in vitro experiments revealed that EB significantly mitigated the reduction in the viability of H9c2 cells, decreased intracellular ROS, downregulated necroptosis-related protein expression, facilitated the nuclear translocation of Nrf2, and upregulated HO-1 in HH-induced H9c2 cells. However, when the Nrf2 inhibitor ML385 was introduced, the beneficial effects of EB on HH-induced H9c2 cells were attenuated. These results suggested that EB inhibited HH-induced necroptosis via the Nrf2-related antioxidation pathway.

In the present study, we emphatically elucidated the anti-necroptosis effect of EB on HAMI. There might be other mechanisms for EB to ameliorate HAMI. Jia et al. [8] reported that pretreatment with eleutheroside E, another bioactive constituent isolated from *Acanthopanax senticosus* (Rupr. et Maxim.) Harms, could alleviate HAMI by regulating NLRP3 inflammasome-mediated pyroptosis. Both necroptosis and pyroptosis are recognized as inflammatory pathways leading to lytic cell death. Mechanistically, activated MLKL initiates NLRP3-dependent caspase-1 activation, suggesting a potential functional connection between these two cell death modalities [42]. Looking ahead, it is worth focusing on clarifying the crosstalk between necroptosis and pyroptosis, as well as exploring the potential of EB to target these two pathways.

## 5. Conclusions

In summary, our research shows that EB improved hypobaric hypoxia-induced oxidative stress, inhibited cardiomyocyte necroptosis, and reduced the levels of inflammatory factors through the Nrf2/HO-1 pathway. Ultimately, myocardial injury induced by hypobaric hypoxia was ameliorated. This study suggested an alternative candidate for the prevention of high-altitude myocardial injury.

## Data Availability

The data used to support the findings of this study are included within the article.

## Abbreviations

AMS	Acute mountain sickness
AS	*Acanthopanax senticosus* (Rupr. et Maxim.) Harms
ANOVA	One-way analysis of variance
BNP	B-type Natriuretic Peptide
CCK-8	Cell Counting Kit-8
CK-MB	Creatine Kinase-MB
EB	Eleutheroside B
ECG	Electrocardiogram
FBS	Fetal bovine serum
GSH	Glutathione
HAMI	High-altitude-induced myocardial injury
HH	Hypobaric hypoxia
HO-1	Heme oxygenase 1
IL-1β	Interleukin-1β
Keap1	Kelch-like epichlorohydrin-related proteins
MDA	Malondialdehyde
MLKL	Mixed-lineage kinase domain-like pseudo kinase
Nrf2	Nuclear factor erythroid 2-related factor 2
OD	Optical density
RIP	Receptor-interacting protein
RIPK1	Receptor-interacting protein kinase 1
RIPK3	Receptor-interacting protein kinase 3
ROS	Reactive oxygen species
TNF-α	Tumor necrosis factor-α
